# Academic Emotion Classification and Recognition Method for Large-scale Online Learning Environment—Based on A-CNN and LSTM-ATT Deep Learning Pipeline Method

**DOI:** 10.3390/ijerph17061941

**Published:** 2020-03-16

**Authors:** Xiang Feng, Yaojia Wei, Xianglin Pan, Longhui Qiu, Yongmei Ma

**Affiliations:** 1 Shanghai Engineering Research Center of Digital Education Equipment, East China Normal University, Shanghai 200062, China; 2 Department of Educational Information Technology, East China Normal University, Shanghai 200062, China; 51194108008@stu.ecnu.edu.cn (Y.W.); 51194101008@stu.ecnu.edu.cn (X.P.); longhuiqiu@126.com (L.Q.); 3 School of Mathematics and Statistics, Chaohu University, Hefei 238000, China

**Keywords:** academic emotion, subjective well-being, academic emotion classification method, academic emotion classification algorithm

## Abstract

Subjective well-being is a comprehensive psychological indicator for measuring quality of life. Studies have found that emotional measurement methods and measurement accuracy are important for well-being-related research. Academic emotion is an emotion description in the field of education. The subjective well-being of learners in an online learning environment can be studied by analyzing academic emotions. However, in a large-scale online learning environment, it is extremely challenging to classify learners’ academic emotions quickly and accurately for specific comment aspects. This study used literature analysis and data pre-analysis to build a dimensional classification system of academic emotion aspects for students’ comments in an online learning environment, as well as to develop an aspect-oriented academic emotion automatic recognition method, including an aspect-oriented convolutional neural network (A-CNN) and an academic emotion classification algorithm based on the long short-term memory with attention mechanism (LSTM-ATT) and the attention mechanism. The experiments showed that this model can provide quick and effective identification. The A-CNN model accuracy on the test set was 89%, and the LSTM-ATT model accuracy on the test set was 71%. This research provides a new method for the measurement of large-scale online academic emotions, as well as support for research related to students’ well-being in online learning environments.

## 1. Introduction

Subjective well-being is a comprehensive psychological indicator that can be used to measure quality of life, and it has an important impact on many aspects, such as people’s health, work status, and social relations [1,2]. An Organization for Economic Co-operation and Development (OECD) report [3] states that children with positive emotions are more likely to grow into happy, confident, and healthy adults, which is of great significance for social development and improvement of people’s well-being. Subjective well-being emerges when people experience a positive emotional experience that is greater than a negative emotional experience [4]. Various studies have used emotion measurement to study its relationship with well-being [5,6,7]. Related studies have shown that positive and negative emotions have significant predictive effects on subjective well-being [8].

Academic emotions are the emotions that students experience in academic situations, and they have received widespread attention in the field of education in recent years. Measuring academic emotions and improving the accuracy of academic emotion measurement serve as a research basis for students’ subjective well-being. Presently, scale-based academic emotion measurement has achieved considerable research results [9,10]. In an online learning environment, the learning platform becomes the main interactive medium. There are a large number of student comments (student comments in this article refer to text-type comments) on the platform, and these comments contain academic emotions. However, it is difficult to apply a scale measurement method for emotions to measure student comments in an online learning environment on a large scale.

With the rapid development of artificial intelligence, using machine learning to automatically identify academic emotions has become a new development trend. For example, convolutional neural networks (CNNs) and long short-term memory (LSTM) are used in sentiment analysis, which improves the accuracy of emotion classification [11,12]. However, there are multiple aspects to student comments in online learning, and students may express emotions for each aspect. The academic emotions expressed on these aspects are better suited for intervention [13]. The following research questions have been raised:

1. What is the overall dimension of student comments in online learning environments? Among them, the aspect and academic emotion dimensions make up the overall dimension. This provides the basis for the realization of aspect-oriented academic emotion analysis.
2. How can an automatic classification and recognition method be developed for aspect-oriented academic emotions?

### 1.1. Subjective Well-being and Academic Emotions

Subjective well-being is the core of the emotional and cognitive evaluation of people’s lives [14]. Subjective well-being usually consists of three components: life satisfaction, positive emotions, and negative emotions [15]. Life satisfaction assesses the cognitive aspect of subjective well-being, while positive and negative emotions assess the emotional experience aspect of subjective well-being [16,17]. Studies have shown that using positive emotion regulation strategies to increase positive emotions and reduce negative emotions can increase subjective well-being [7,8,18,19]. Subjective well-being depends on the emotional experience obtained by regulating emotions [20]. Regardless of whether the regulation comes from student self-regulation or external intervention, the basic premise is to recognize the types of emotions currently being expressed and the aspects of the emotions.

Academic emotion is one of the subordinate concepts of emotion, which has become a popular issue in foreign pedagogy and psychology research. Pekrun et al. [21] pointed out that academic emotions refer to various emotional experiences related to students’ academic activities during teaching and learning. Pekrun et al. [13] further expanded the definition of academic emotions to include the various emotions that students experience regarding academic success or failure, as well as emotional experiences during classroom studies, daily homework assignments, and exams; they also proposed different types of academic emotions.

Academic emotions are a kind of nonintellectual factor closely related to the teaching and learning process, which play an important role in students’ learning processes. Many studies have found that academic emotions affect students’ psychological flexibility, self-regulated learning, and academic performance [22,23,24], of which positive academic emotions can enhance intrinsic motivation [25] and predict students’ academic performance [26], while negative academic emotions predict negative academic performance [27]. In addition, academic emotions also affect students’ mental health and, thus, students’ subjective well-being [28].

Students have many emotional experiences in online learning environments which affect their study, performance, interaction, and well-being. However, due to the limitations of the physical environment, students’ emotions cannot be directly observed. The measurement of academic emotions has become an important research area in online learning environments. Therefore, measuring academic emotions is a pertinent basis for research on students’ subjective well-being.

### 1.2. Measurement and Recognition of Academic Emotion

Most current research measures academic emotions based on an academic emotion scale. Pukrun et al. [21] developed an academic emotion questionnaire to measure college students’ academic emotions. Based on the theory of achievement emotion control value proposed by Pekrun, Pekrun et al. [29] proposed the revision of the Achievement Emotions Questionnaire (AEQ) in 2011. Lichtenfel et al. [30] proposed the Achievement Emotions Questionnaire—Elementary School (AEQ-ES) in 2012 considering the three dimensions of happiness, anxiety, and boredom. Several studies investigated the relationship between emotions and mental health or well-being based on an emotional scale [31]. Wang et al. [32] explored the relationship between negative academic emotions, mental health, and cognitive re-evaluation through a questionnaire survey.

However, these academic emotion measurement methods are difficult to apply to large-scale student comments in an online learning environment. With the development of artificial intelligence technology in the field of education, sentiment analysis technology has been gradually applied to academic emotion analysis of student comments [33,34], so that the emotions expressed by students in texts can be automatically extracted. Pang et al. used Naive Bayes (NB), maximum entropy classification, and support vector machines (SVMs), which are three machine learning methods, for sentiment classification, and the results were worse than traditional topic-based categorization [35]. Kim applied a CNN, which performs well in image processing, to text emotion analysis and achieved a good classification effect [36]. Shamsi et al. applied K-Means, Fuzzy C-Means (FCM), and other different types of clustering technology to data describing human emotions, providing an effective method for an online comment classification system based on Twitter, blog, and social media content [37]. In recent years, with the in-depth study of attention mechanisms, it has been widely used in popular fields such as natural language processing. The Self Attention mechanism proposed by Google’s machine translation team has received widespread interest, as it provided state-of-the-art results on both the ACL 2014 Ninth Workshop on Statistical Machine Translation (WMT 2014) English-to-German and WMT 2014 English-to-French translation tasks [38]. Feng et al. [39] established an academic emotion recognition model based on LSTM in deep learning, which realized automatic recognition of academic emotions, proving that this is a feasible research route. In summary, we found that a CNN model can extract sentence features more effectively, thereby improving the accuracy of sentiment analysis. The Self Attention mechanism can effectively capture the internal structure of sentences, and the bi-LSTM model can better learn the bidirectional semantic dependence of a text.

Early academic emotion classification learned from the classification of emotion and divided academic emotions into positive and negative dimensions. Patrick et al. [40] divided children’s emotional experiences in learning activities into positive emotions (interest, happiness, and relaxation), boredom, pain, and anger after studying the academic emotions experienced in learning activities during childhood. However, simply distinguishing academic emotions from positive and negative states cannot meet the actual required needs. In the process of analyzing academic emotions, researchers also need to pay attention to the activated states of emotions. Therefore, Pekrun et al. [29] divided academic emotions into four dimensions—positive activating, positive deactivating, negative activating, and negative deactivating—according to the degree of happiness and activation of students in the learning process.

Academic emotions in student comments have attracted widespread attention from researchers. Existing studies directly mined academic emotions from online student comment texts, ignoring the complexity of online course comment texts. For example, students may feel frustrated because the webpage is too slow, or they may feel happy because the learning activities are interesting. In addition to containing a variety of emotions or opinions, there are different aspects to student comments. The aim of this study was to use machine learning methods to automatically identify students’ academic emotions on different aspect categories. Here, we have explained the background of the research, established the overall dimension of student comments in an online learning environment, and built a pipeline method for automatic identification of academic emotions based on the established dimensions. Based on the aspect dimension, an aspect-oriented convolutional neural network (A-CNN) (Supplementary Document S1) classification model was constructed on the basis of a CNN. Based on the dimensions of academic emotions, an academic emotion classification long short-term memory with attention mechanism (LSTM-ATT) (Supplementary Document S2) model was constructed on the basis of the LSTM model combined with the attention mechanism.

## 2. Materials and Methods

### 2.1. Aspect-oriented Academic Emotion Classification Method for Online Learning Platforms

#### 2.1.1. Overall Dimension

The overall dimension was composed of aspect and academic emotion dimensions (Table 1). The aspect dimension is the object of emotional expression in the student comments, and the academic emotion dimension is the specific academic emotion contained in the comment text. These two dimensions are explained in Section 2.1.2 and Section 2.1.3, respectively.

#### 2.1.2. Aspect-oriented Dimension

**Figure 1:**
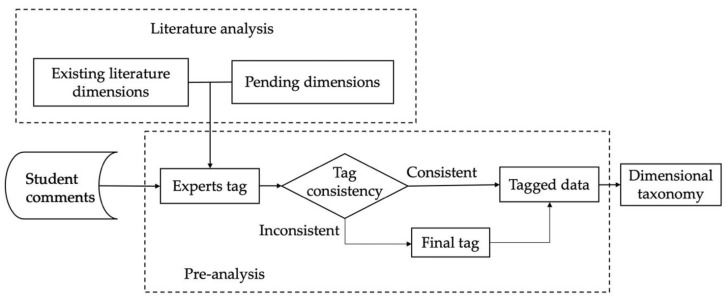
Aspect-oriented academic emotion classification system.

In this study, firstly, based on the existing literature, the required dimensions were extracted by using a literature analysis as a reference, and then the final aspect classification system was established by the expert annotation preanalysis method. The aspect-oriented academic emotion classification system is shown in Figure 1.

In previous research, the evaluations proposed in online learning platforms have been diverse, but the evaluation dimensions of online learning platform comments have not been given enough attention. In [41], they used text mining technology, combined with existing curriculum evaluation standards [42,43], and used statistical analysis to select indicators that are valued by most authoritative methods; then, they established an evaluation model based on the Massive Open Online Course (MOOC) curriculum. The model mainly pointed out five dimensions: instructional design, course content, course design, interface design, and media technology. This study used five dimensions as the basic dimensions of the comment text analysis and established the final dimensions by preanalyzing the data.

Based on these five basic aspect dimensions, a crawler program was used to obtain student comments from online learning platforms (Tencent Class and China University MOOC) [44,45]. After cleaning the data, the comment texts were preprocessed. During preprocessing, three experts tagged each comment text to ensure the accuracy of data tagging. The final establishment of the data-tagged dimension was based on the tagged results of the three persons. If the three persons’ tagged results were consistent, the dimension of this comment text was the tagged result of the three persons. If the three persons’ tagged results were inconsistent, a final tagged result was constructed to decide the comment text. If the comment text aspects were not related to the five aspects, it was tagged as an unassured dimension comment text.

Through manual preanalysis of these tagged data, it was found that the data amount of the interface design, media technology, course design, and course content was too small, so it was not universal. We considered the following: (1) the Education Informatization Standard “Course Evaluation Standards for Online Courses (CELTS-22) [46]“ drafted by the Ministry of Education committee; (2) the online course evaluation standards in previous research, interface design, and media technology were combined into the online learning platform dimension, and course design and curriculum management were combined into the course dimension; (3) the subsequent training of the data using neural network models would cause these categories with less data to be significantly less accurate in prediction than those with large training data. Therefore, a model for classifying academic emotion aspects was constructed, and the student comments were mainly divided into three categories: teacher, course, and online learning platform dimensions.

#### 2.1.3. Academic Emotion Dimension

Based on the classification methods of academic emotions in previous research [39,47,48,49], this study integrated the academic emotion category of college students and adolescents and constructed the dimension as a secondary dimension. The first dimension included four categories: positive activating, positive deactivating, negative activating, and negative deactivating. The second dimension included nine categories: enjoyment, hope, joy, relaxation, anger, shame, anxiety, disappointment, and boredom.

### 2.2. Aspect-oriented Academic Emotion Classification Algorithm Based on A-CNN and LSTM-ATT

#### 2.2.1. Academic Emotion Automatic Recognition Framework Oriented to Aspect Categories

**Figure 2:**
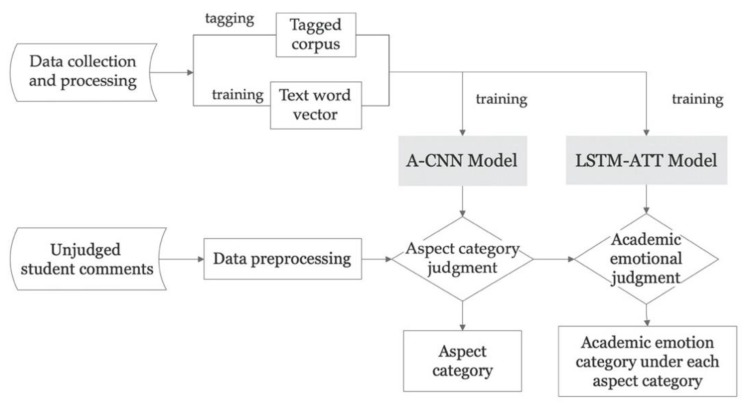
Academic emotion automatic recognition pipeline framework.

This study designed a pipeline architecture of aspect-oriented academic emotion automatic recognition, including the aspect classification model A-CNN and the academic emotion classification model LSTM-ATT (Figure 2). A-CNN was proposed based on the basic model of a CNN. LSTM-ATT combines the LSTM model and the attention mechanism. After data preprocessing, the student comment text was firstly input into the A-CNN network model to judge the aspect category. After the results were saved, the academic emotion category was judged by the LSTM-ATT network model. Finally, the aspect (object of emotion) of the different comment texts and the academic emotions (emotion category) under the aspect category were obtained.

#### 2.2.2. The A-CNN Model

**Figure 3:**
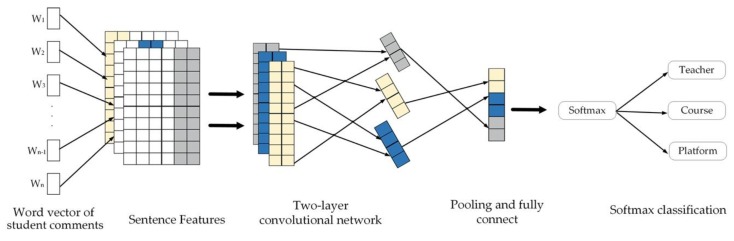
The aspect-oriented convolutional neural network (A-CNN) model.

The A-CNN’s text classification results were divided into the three dimensions of aspect categories, namely, teachers, course, and online learning platform. In previous studies, CNNs have not only made great achievements in image processing but have also been proved to be effectively applied to natural language processing tasks, such as text classification [36] and semantic interpretation [50]. The basic CNN model is mainly composed of a convolutional layer, a pooling layer, and a fully connected layer. The network structure of the A-CNN includes a sentence feature layer, two CNN layers, a fully connected layer, and a softmax classification layer (Figure 3).

After preprocessing the comment text, a word segmentation tool was used to decompose each sentence into a vocabulary, and the word vector training tool was used in the sentence feature layer to convert the vocabulary in the sentence into a word vector representation. Sentence features are composed of a single word in the sentence, such as sentence S1 = [“讲的真心不错，希望以后多一点课 (The teaching is really good, hope to have more classes in the future)”], sentences after word segmentation Segment-S1 = [“讲 (The teaching)”,“的 (is)”,“真心 (really)”,“不错 (good)”, “，”,“希望 (hope)”,“以后 (in the future)”,“多一点 (to have more)”,“课 (class)”]; the feature of S1 is represented by the set of each lexical feature after sentence segmentation. In the sentence feature layer, by combining the features of vocabulary in the sentence, the number of features of the sentence increases greatly, and the feature composition of each sentence is inconsistent. $W_{i}$ represents the sentence word vector of the *i*th word in the sentence, $W_{i}\in R_{k}$, where k represents the dimension of the vector. Therefore, in this work, we defined the sentence vector $S_{\mathrm{ve}c\mathrm{tor}}$ (Formula (1)), where n represents the length of the sentence, $W_{i}\left(i\in \left[1,n\right]\right)$ represents the vector of the *i*th term in the sentence, and “+” represents the join operation of the vector. The sentence represented by the word vector feature was mapped to the matrix Mk*n of k*n in space and then input into the CNN network layer.

After two layers of CNN convolution operation, the features in the sentence achieved effective dimensionality reduction. In the two-layer CNN network, each CNN network layer contained multiple convolution kernels, each of which had a fixed size of K=2c+1. Then, Formula (2) was executed for the convolution operation, and the output result used the ReLU activation function. Among them, $V_{i,j}$ represents the element value of the position of the convolution kernel corresponding to the output matrix, M_n is the number of input matrices, $X_{k}$ is the *k*th input matrix, $W_{k}$ is the *k*th convolution kernel matrix, and *b* is the offset.

Then, the output results of the two-layer convolutional network were collected to the MAX pooling layer to further reduce the dimension of features. Next, we applied a fully connected layer, obtained weights after all locations shared training, and output the results to the softmax layer. The softmax layer mapped the input to the interval (0,1) through the softmax function to obtain the probability that the data to be classified belonged to various categories and finally obtained the prediction result of the aspect category.

(1) $$S_{vector}=W_{1}+W_{2}+\cdots +W_{n},$$

(2) $$V_{i,j}={\left(X*W\right)}_{i,j}+b=\overset{M\_n}{\underset{k=1}{\sum }}{\left(X_{k}+W_{k}\right)}_{i,j}+b,$$

#### 2.2.3. The LSTM-ATT Model

**Figure 4:**
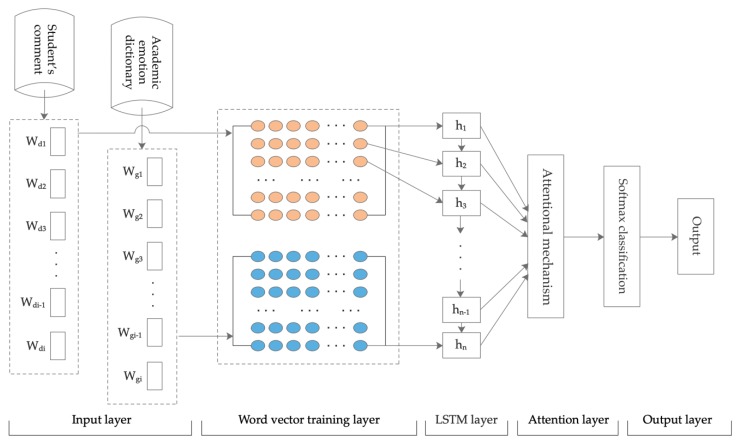
The long short-term memory with attention mechanism (LSTM-ATT) network model.

The classification results of the LSTM-ATT relate to nine categories of academic emotion, including enjoyment, hope, joy, relaxation, anger, shame, anxiety, disappointment, and boredom. The team’s previous research results confirmed that the use of LSTM network training models can predict academic emotion [39]. In recent years, the attention mechanism has been applied to emotion analysis tasks. The attention mechanism is a focus distribution model. For specific tasks, the focus of attention can be on some features, so as to increase the weight of the focus part and improve the influence of the focus part on the whole, while the nonfocus part can be ignored. The practical application of the attention mechanism can be divided into two categories: soft attention and hard attention. For soft attention, in the process of calculating the attention weight, all data are included in the attention range, and no filter conditions for the data feature are set. Hard attention sets the filtering condition after calculating the attention weight and sets a part of the attention weight value that does not meet the condition to 0. In this study, we used the soft attention mode for self-attention of the data features of student comments. LSTM-ATT mainly has five layers: input, word vector training, LSTM network training, attention, and output layers (Figure 4).

Input layer. The input layer contained two kinds of data: The first was the student comments which had been judged by the aspect category; The second was a dictionary of academic emotions. In Figure 4, $W_{d}$ represents the vocabulary data set of student comments, $W_{di}$ represents the *i*th vocabulary in the set, $W_{g}$ represents the academic emotion dictionary, and $W_{gi}$ represents the *i*th vocabulary in the set.

The research in this paper shows that the classification accuracy can be improved by using the academic emotion dictionary as the basis for the classification of academic emotions. The construction method of the academic emotion dictionary adopted the automatic construction method. The first step was to establish a multidimensional vector corpus. Preprocessing was performed for the student comment corpus, including Chinese word segmentation and removal of the stopped words of the text, and then the Word2vec [51] tool was used to convert the preprocessed corpus into a word vector. The second step was to expand candidate words based on the multidimensional vector corpus. By using the vector database of student comment words, we were able to automatically select emotion words from the seed vocabulary and add them to the candidate words set, so as to expand the candidate emotion words. The third step was to classify the candidate emotion vocabulary and add it to the dictionary of corresponding academic emotion classification.

Word vector training layer. The vocabulary set of two corpora was input and converted into a 300-dimensional vector in the vector training layer. Each word vector had a unique matrix representation.

LSTM network training layer. The LSTM model was the core layer of the academic emotion classification model. Multiple LSTM units were set in this layer. The number of cells of LSTM was consistent with the number of time steps. All fusion vectors entered the LSTM cell, and when a time step was completed, the value of the hidden layer of each cell was output, and then the fusion vector entered the next time step.

**Figure 5:**
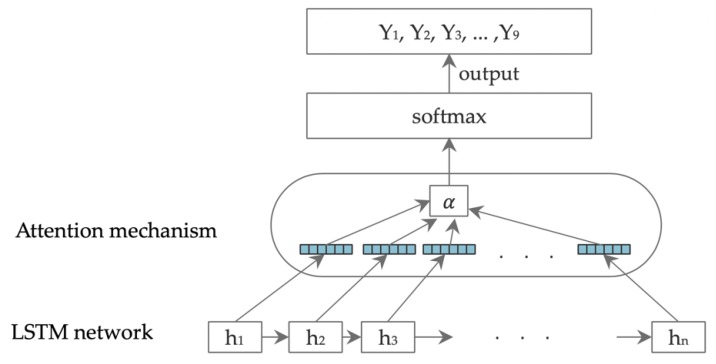
LSTM hidden layer to attention mechanism flow description.

Attention layer. The hidden layer output vectors of all LSTM networks H=[h1,h2,h3⋯ht], where t represents the length of the output, as shown in Figure 5.

Output layer. The output results of the attention layer were used in the output layer, and the values in the range of [0, 1] were output using the activation function. Each output result contained nine dimensions of academic emotions, corresponding to the probability of nine categories. The index value with the highest probability was the prediction category. Under the pipeline framework, the same student comments would judge the aspect category and then judge the academic emotion; finally, the aspect category and the academic emotion category of different texts were obtained.

### 2.3. Experiment

#### 2.3.1. Data Preparation

In this study, a crawler program was used to obtain more than 200,000 records on online learning platforms for student comments, including 130,000 records from “腾讯课堂” [44], 70,000 records from “中国大学MOOC” [45], and 8000 blogs of students’ feelings.

The data preprocessing mainly included Chinese word segmentation and removing special characters and stopping words. First, special characters and stop words were removed to reduce the influence of experimental results. Second, Chinese word segmentation was performed. In the field of natural language processing, words and phrases in Chinese text appear continuously without obvious segmentation marks. In this study, Jieba was used as a word segmentation tool [52].

After data preprocessing, aspect category tagging and academic emotion tagging was carried out, so as to construct a corpus of academic emotion analysis. Tagged data for aspects were stored as a binary group: < text content, aspect tagged dimension >. Tagged data of academic emotions were also stored in the form of binary groups: < text content, academic emotion tagged dimension >. For example, the aspect dimension of the text “徐老师讲课认真负责，也很好理解，对学习很有帮助 (Mr. Xu is conscientious and responsible in class, and the content is well understood, which is very helpful for learning)” is “teacher”, and the academic emotion dimension is “joy”. The aspect dimension is saved in the form of < “徐老师讲课认真负责，也很好理解，对学习很有帮助 (Mr. Xu is conscientious and responsible in class, and the content is well understood, which is very helpful for learning)”, teacher >, and the academic emotion dimension is preserved in the form of < “徐老师讲课认真负责，也很好理解，对学习很有帮助 (Mr. Xu is conscientious and responsible in class, and the content is well understood, which is very helpful for learning)”, joy >. After many people participated in the data tagging, we obtained a total of 16,925 pieces of text data. Among them, there were 8213 items of aspect-tagged data and 8712 items of academic-emotion-tagged data. During the experiment, the ratio of training set data to verification set data was 8:2.

**Figure 6:**
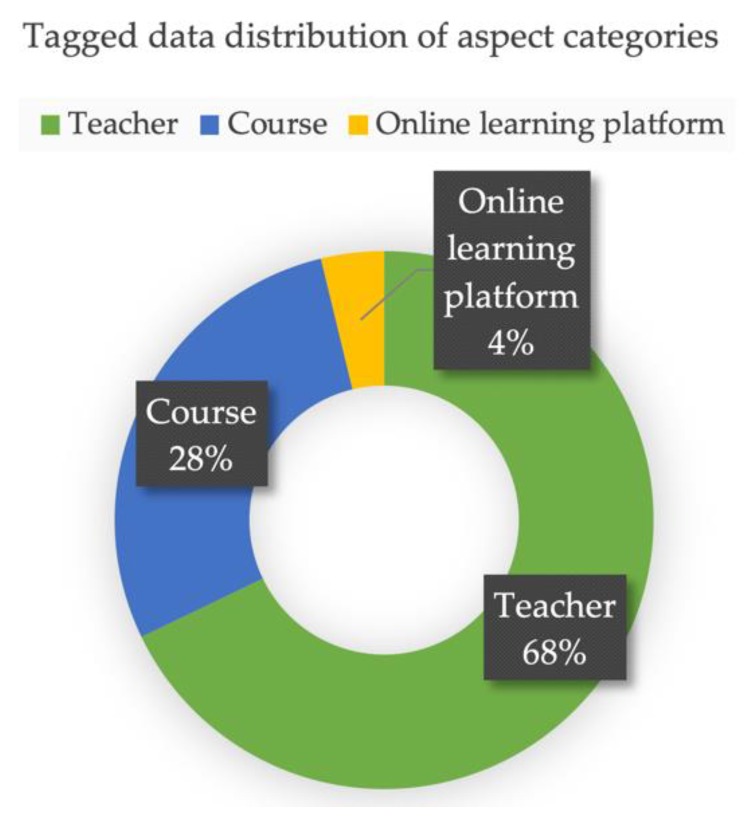
Tagged data distribution picture of aspect categories.

The tagged data distribution of the aspect categories is shown in Figure 6. From the perspective of distribution, the majority of emotional expression objects of online learning student comment texts were concentrated in the teacher dimension, accounting for 68%.

**Figure 7:**
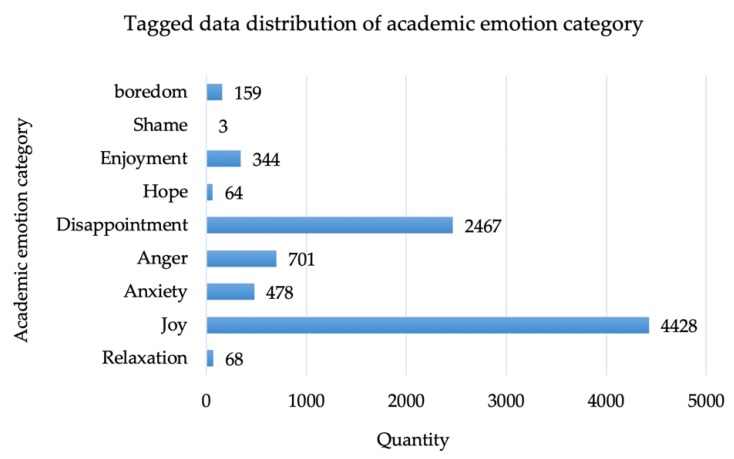
Tagged data distribution picture of academic emotion category.

The tagged data distribution of the academic emotion category is shown in Figure 7. According to the distribution of academic emotion categories, academic emotions were concentrated in the “joy” and “disappointment” categories in most online learning comment texts, accounting for 79%.

#### 2.3.2. Word Vector Training

Using the Word2vec tool [51], the corpus of subject and academic emotion tagging was transformed into a word vector. When deep learning was used for text classification task, it was necessary to convert semistructured or unstructured text into a vector representation that could be understood and processed by the computer.

In the training process of the Word2vec word vector, we chose the best parameter result through multiple rounds of iterative tuning based on the size of the experimental data set. The specific parameters in the word vector training process were set as follows: vector length vector_size = 300, window size window = 5, model was NegativeSampling Skip-gram model, neural network learning rate alpha = 0.025, word frequency min_count was set to 3, and number of training iterations was 8.

#### 2.3.3. A-CNN and LSTM-ATT Training

In this experiment, A-CNN parameters were obtained by iterative and mutual optimization. They were set to loss function loss = “binary_crossentropy”, optimizer = “adam”, batch_size = 32, and epochs = 4.

The LSTM-ATT used bidirectional long short-term memory (BiLSTM) and soft attention. The specific parameters in LSTM-ATT were set to batch_size = 32, epochs = 4, return_sequences = True, and units = 100.

## 3. Results

**Figure 8:**
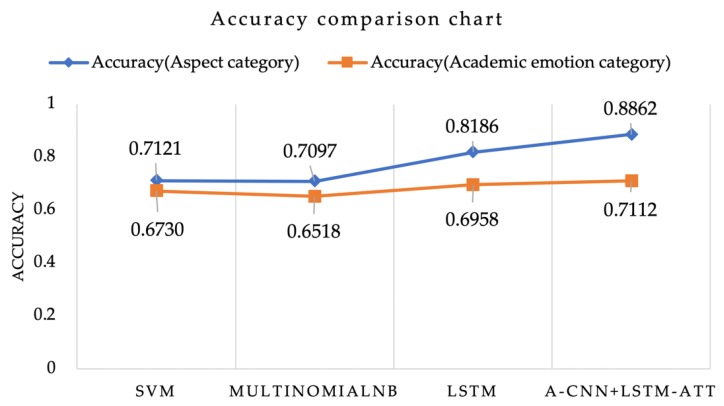
Accuracy comparison of each model.

In this study, 1643 aspect-tagged data and 1742 academic emotion-tagged data were used to test the above-described training model. Through the experiment, compared with SVM, MultinomialNB, and LSTM prediction accuracy, the aspect classification accuracy of A-CNN was 88.62%, and that of LSTM-ATT was 71.12% (Figure 8).

**Figure 9:**
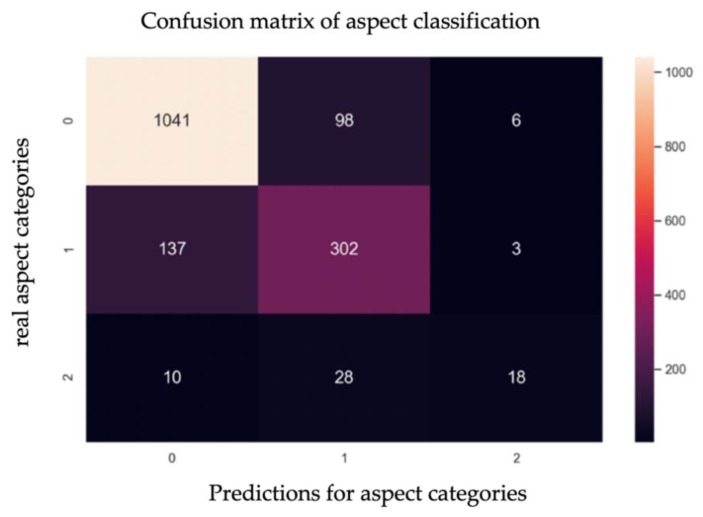
Confusion matrix of aspect classification.

The normalized confusion matrix of the aspect category prediction results is shown in Figure 9. The label “0” indicates the “teacher” category, “1” indicates the “course” category, and “2” indicates the “platform” category. It can be seen from the confusion matrix that the accuracy rate of data prediction for “teacher” reached 90.92%. In the categories of “course” and “platform”, because the training data set occupied a relatively small proportion in the overall training data, accounting for 28% and 4%, respectively, the model had relatively low discrimination between the two types of data.

**Figure 10:**
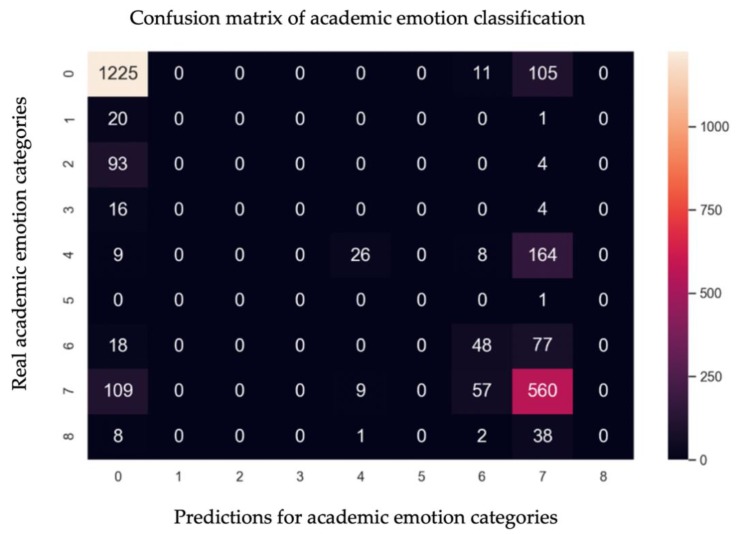
Confusion matrix of academic emotion classification.

The normalized confusion matrix of academic sentiment prediction results is shown in Figure 10. The labels “0–8” indicate joy, hope, enjoyment, relaxation, anger, shame, anxiety, disappointment, and boredom, respectively. In the model prediction confusion matrix, the data of the emotion categories “joy” and “disappointment” had higher prediction results, with accuracy rates of 91.35% and 76.19%. This was because during the training of the model, the data were concentrated in the “joy” and “disappointment” categories, so it could better identify the academic emotional features contained in such text.

## 4. Discussion

This paper proposes a kind of academic emotion analysis technology based on artificial intelligence methods for online learning which can help researchers to conduct research on learner well-being based on academic emotions. In previous studies, the Achievement Emotion Questionnaire [29] was the most commonly used emotion survey in educational settings [53]. However, this method is not suitable for large-scale online learners. Students’ academic emotions have an impact on their academic performance, mental health, and other aspects, thus influencing their learning satisfaction and well-being [22,23,24,28,54]. Future research can be based on the recognition results of this algorithm, on the development of a large-scale online-learning-environment-oriented massive investigation and study, and on trying to understand students’ well-being and its mechanism in online learning environments through big data analysis methods and ideas. Thus, for large-scale online learning platforms and courses, this method provides valuable results about student well-being.

Limitations of the research: The academic emotion analysis technology based on deep learning requires a large amount of training data as a support during the model training process. Regarding the amount of tagged data, there was a large difference in the distribution of each category of data. Some categories of data accounted for a small amount, but they actually existed in the text of student comments. In the aspect-tagged data, there were 5573 entries for the “teacher” category and 310 entries for the “platform” category. Therefore, when using the A-CNN model to train the data, the category with a lesser amount of data was significantly lower in the accuracy of prediction than the category with a larger amount of training data. In future work, the labeling of corpus resources should be better suited to label the less frequent category data, so that the data distribution of each training category is more balanced, and the accuracy of academic emotion analysis is further improved. Text-based emotion analysis is a research topic that has received widespread attention. Research results for various specific fields are continuously being produced. Since this research mainly focused on application-level exploration and was limited by the time and manpower of the research, we did not compare it with state-of-the-art algorithms but with the basic SVM and LSTM.

In addition, although this study achieved some results in the aspect classification of student comments, it divided the aspects expressed in student comments into teacher, course, and platform, so as to know the aspect category of emotional expression. However, this approach is still essentially categorizing student text into known categories. In student comments, though, some of the emotional expression of the text is the subject of a knowledge point or a specific thing, for example, learner autonomy [55] and students’ interaction patterns in online discussions [56]. These require a more detailed aspect extraction method to customize the extraction of all aspect categories in the text for a more granular emotional analysis of student comments. Therefore, in future work, we will focus more on the extraction of aspects from students’ comments, so as to make the automatic analysis of academic emotions more targeted.

## 5. Conclusions

In an online learning environment, teachers and course builders need to understand what kind of academic emotions students have developed for which aspect in order to undertake necessary and effective interventions to improve the well-being of students in the online learning environment. According to the academic emotion tagged data, we can see that in the distribution of aspect classification, the largest proportion of student comments was for teachers, followed by the course. We know that the quality of teachers is important for the well-being of students. From the distribution of academic emotion classification, academic emotions were mainly concentrated in the “joy” and “disappointment” dimensions. The classification of academic emotions can better support related research on academic emotion calculation and can help teachers and course developers predict public demand, which can promote prompt and effective responses and help increase the well-being of teachers and students. In order to accurately and quickly obtain the academic emotions contained in the comments and their corresponding aspects, effective automated methods need to be developed. To solve this problem, based on deep learning technology, we built a framework of student comment aspect classification and academic emotion classification models. Based on this, a machine learning data set was produced, and then an analysis framework of A-CNN and LSTM-ATT fusion was developed. The experimental results showed that the student comment aspect classification model and the academic emotion classification model proposed in this paper are superior to general machine learning models and conventional LSTM network models. The accuracy of the aspect classification model was 88.62% and that of the academic emotion classification model was 71.12%.

## Figures and Tables

**Table 1:** Overall dimension.

Aspect Dimension	Academic Emotion Dimension	
	First Dimension	Second Dimension
Teacher, Course, Online learning platform	positive activating	enjoyment, hope, joy
	positive deactivating	relaxation
	negative activating	anger, anxiety, shame
	negative deactivating	disappointment, boredom

## Supplementary Materials

The following are available online at https://www.mdpi.com/1660-4601/17/6/1941/s1, Document S1: The core code of A-CNN, Document S2. The core code of LSTM-ATT.
