# Supplementary material for: Academic Emotion Classification and Recognition Method for Large-scale Online Learning Environment—Based on A-CNN and LSTM-ATT Deep Learning Pipeline Method
Source: Int J Environ Res Public Health. 2020 Mar 16;17(6):1941. doi: 10.3390/ijerph17061941 (PMC7142864; doi:10.3390/ijerph17061941)
Supplement: Supplementary file 1 [file ijerph-17-01941-s001.pdf]

**Supplementary materials:**

**Document S1.** The core code of A-CNN.

```
# take tokens and build word-id dictionary
tokenizer = Tokenizer(filters='!"#$%&()*+,-./:;<=>?@[\\]^_`{|}~\t\n',lower=True,split=" ")
tokenizer.fit_on_texts(X)
vocab = tokenizer.word_index

# Load word vector
word2vec_path = '../../trainWord2vec/vectorModel/word2vec.model'
word2vec_model = gensim.models.Word2Vec.load(word2vec_path)

# Match the word vector for each word in the data set from Glove
embedding_matrix = np.zeros((len(vocab) + 1, 300))
for word, i in vocab.items():
    try:
        embedding_vector = word2vec_model[word]
        embedding_matrix[i] = embedding_vector
    except:
        pass



# Match the input format of the model
x_train_word_ids = tokenizer.texts_to_sequences(x_train)
x_test_word_ids = tokenizer.texts_to_sequences(x_test)
x_train_padded_seqs = pad_sequences(x_train_word_ids, maxlen=30)
x_test_padded_seqs = pad_sequences(x_test_word_ids, maxlen=30)


# TextCNNBN
main_input = Input(shape=(30,), dtype='float64')
embedder = Embedding(len(vocab) + 1, 300, input_length = 30, weights = [embedding_matrix], trainable
= False)
embed = embedder(main_input)

conv1_1 = Convolution1D(256, 3, padding='same')(embed)
bn1_1 = BatchNormalization()(conv1_1)
relu1_1 = Activation('relu')(bn1_1)
conv1_2 = Convolution1D(128, 3, padding='same')(relu1_1)
bn1_2 = BatchNormalization()(conv1_2)
relu1_2 = Activation('relu')(bn1_2)
cnn1 = MaxPool1D(pool_size=4)(relu1_2)

conv2_1 = Convolution1D(256, 4, padding='same')(embed)
```

```
bn2_1 = BatchNormalization()(conv2_1)
relu2_1 = Activation('relu')(bn2_1)
conv2_2 = Convolution1D(128, 4, padding='same')(relu2_1)
bn2_2 = BatchNormalization()(conv2_2)
relu2_2 = Activation('relu')(bn2_2)
cnn2 = MaxPool1D(pool_size=4)(relu2_2)

conv3_1 = Convolution1D(256, 5, padding='same')(embed)
bn3_1 = BatchNormalization()(conv3_1)
relu3_1 = Activation('relu')(bn3_1)
conv3_2 = Convolution1D(128, 5, padding='same')(relu3_1)
bn3_2 = BatchNormalization()(conv3_2)
relu3_2 = Activation('relu')(bn3_2)
cnn3 = MaxPool1D(pool_size=4)(relu3_2)

cnn = concatenate([cnn1,cnn2,cnn3], axis=-1)
flat = Flatten()(cnn)
drop = Dropout(0.5)(flat)
fc = Dense(512)(drop)
bn = BatchNormalization()(fc)
main_output = Dense(num_labels,activation='softmax')(drop)
model = Model(inputs = main_input, outputs = main_output)

model.compile(loss='binary_crossentropy',
              optimizer='adam',
              metrics=['accuracy'])

history = model.fit(x_train_padded_seqs, y_train,
                    batch_size=32,
                    epochs=4,
                    validation_data=(x_test_padded_seqs, y_test))
```

**Document S2.** The core code of LSTM-ATT.

```
#data preparation
def datapPepare(path):

    lexiconFile = pd.read_excel(path)
    lexicon = []
    lexiconArray = lexiconFile.lexicon
    for word in lexiconArray:
        lexicon.append(word)
    return lexicon

if __name__=='__main__':



    # ------ data preparation -------
    lexicon = datapPepare('./lexicon/emotionLexicon.xls')
    df = pd.read_csv('./trainData/emotionData.csv')
    content = df.content
    polar = df.polar

    words = []
    for con in content:
        cut = list(jieba.cut(con.strip(), cut_all=False))
        sentence = ''
        for one in cut:
            if one in lexicon:
                sentence = sentence + one + ' '
                # print(sentence)
        for word in cut:
            sentence = sentence + word + ' '

        words.append(sentence)

    dataframe = pd.DataFrame({'content': words, 'polar': polar})
    dataframe.to_csv("./trainData/emotionDataTrain.csv", index=False, sep=',')
    print('emotionDataTrain.csv 完成')
    df = pd.read_csv('./trainData/emotionDataTrain.csv')
    X = df.content
    label = df.polar

    x_train, x_test, y_train, y_test = train_test_split(X, label, test_size=0.3, random_state=19)

    y_labels = list(y_train.value_counts().index)
```

```
le = preprocessing.LabelEncoder()
le.fit(y_labels)
num_labels = len(y_labels)
y_train = to_categorical(y_train.map(lambda x: le.transform([x])[0]), num_labels)
y_test = to_categorical(y_test.map(lambda x: le.transform([x])[0]), num_labels)

tokenizer = Tokenizer(filters='!"#$%&()*+,-./:;<=>?@[\\]^_`{|}~\t\n', lower=True, split=" ")
tokenizer.fit_on_texts(X)
vocab = tokenizer.word_index

word2vec_path = '../trainWord2vec/vectorModel/word2vec.model'
word2vec_model = gensim.models.Word2Vec.load(word2vec_path)

# Match the word vector for each word in the data set from Glove
embedding_matrix = np.zeros((len(vocab) + 1, 300))
for word, i in vocab.items():
    try:
        embedding_vector = word2vec_model[word]
        embedding_matrix[i] = embedding_vector
    except:
        pass

x_train_word_ids = tokenizer.texts_to_sequences(x_train)
x_test_word_ids = tokenizer.texts_to_sequences(x_test)
x_train_padded_seqs = pad_sequences(x_train_word_ids, maxlen=30)
x_test_padded_seqs = pad_sequences(x_test_word_ids, maxlen=30)


# -------attention mechanism-----------
class AttLayer(Layer):
    def __init__(self, init='glorot_uniform', kernel_regularizer=None,
                 bias_regularizer=None, kernel_constraint=None,
                 bias_constraint=None, **kwargs):
        self.supports_masking = True
        self.init = initializers.get(init)
        self.kernel_initializer = initializers.get(init)

        self.kernel_regularizer = regularizers.get(kernel_regularizer)
        self.bias_regularizer = regularizers.get(kernel_regularizer)

        self.kernel_constraint = constraints.get(kernel_constraint)
        self.bias_constraint = constraints.get(bias_constraint)

        super(AttLayer, self).__init__(**kwargs)
```

```
def build(self, input_shape):
    assert len(input_shape) == 3
    self.W = self.add_weight((input_shape[-1], 1),
                             initializer=self.kernel_initializer,
                             name='{}_W'.format(self.name),
                             regularizer=self.kernel_regularizer,
                             constraint=self.kernel_constraint)
    self.b = self.add_weight((input_shape[1],),
                             initializer='zero',
                             name='{}_b'.format(self.name),
                             regularizer=self.bias_regularizer,
                             constraint=self.bias_constraint)
    self.u = self.add_weight((input_shape[1],),
                             initializer=self.kernel_initializer,
                             name='{}_u'.format(self.name),
                             regularizer=self.kernel_regularizer,
                             constraint=self.kernel_constraint)

    self.built = True

def compute_mask(self, input, input_mask=None):
    return None

def call(self, x, mask=None):
    uit = K.dot(x, self.W)  # (x, 40, 1)
    uit = K.squeeze(uit, -1)  # (x, 40)
    uit = uit + self.b  # (x, 40) + (40,)
    uit = K.tanh(uit)  # (x, 40)

    ait = uit * self.u  # (x, 40) * (40, 1) => (x, 1)
    ait = K.exp(ait)  # (X, 1)

    if mask is not None:
        mask = K.cast(mask, K.floatx())  # (x, 40)
        ait = mask * ait  # (x, 40) * (x, 40, )

    ait /= K.cast(K.sum(ait, axis=1, keepdims=True) + K.epsilon(), K.floatx())
    ait = K.expand_dims(ait)
    weighted_input = x * ait
    output = K.sum(weighted_input, axis=1)
    return output

def compute_output_shape(self, input_shape):
```

```
        return (input_shape[0], input_shape[-1])



#--------LSTM model training-------------

inputs = Input(shape=(30,), dtype='float64')
embed = Embedding(len(vocab) + 1, 300, input_length=30, weights=[embedding_matrix], trainable=False)(inputs)
l_lstm = Bidirectional(LSTM(100, return_sequences=True))(embed)
l_att = AttLayer()(l_lstm)
output = Dense(num_labels, activation='softmax')(l_att)
model = Model(inputs, output)

model.compile(loss='categorical_crossentropy',
              optimizer='adam',
              metrics=['accuracy'])

tb = TensorBoard(log_dir='log/',
                 histogram_freq=1,
                 batch_size=32,
                 write_graph=True,
                 write_grads=False,
                 write_images=False,
                 embeddings_freq=0,
                 embeddings_layer_names=None,
                 embeddings_metadata=None)
callbacks = [tb]

history = model.fit(x_train_padded_seqs, y_train,
          batch_size=32,
          epochs=4,
          validation_data=(x_test_padded_seqs, y_test),callbacks = callbacks)
```
